# Gustatory dysfunction perceptions versus objective gustatory dysfunction among older adults

**DOI:** 10.1186/s12877-023-03781-w

**Published:** 2023-01-31

**Authors:** Seung Yong Park, Kyung Soo Kim, Hyun Jin Min

**Affiliations:** Department of Otorhinolaryngology-Head and Neck Surgery, Chung-Ang University College of Medicine, 102, Heukseok-ro, Dongjak-gu, Seoul, 156-755 South Korea

**Keywords:** Geriatric, Subjective, Measured, Gustation, Olfaction

## Abstract

**Background:**

Research on gustatory dysfunction among older adults has been scarce relative to research on olfactory dysfunction, and the relationship between subjective and objective gustatory dysfunction has not been studied in detail. We aimed to evaluate whether subjective recognition of gustatory dysfunction correlates with objective diagnosis of gustatory dysfunction among older adults.

**Methods:**

In this retrospective, cross-sectional study, we reviewed the medical records of 138 patients of ages ≥ 60 years for whom data were available on self-reported and objectively measured gustatory and olfactory function from January 2018 through April 2021 at a specialized smell/taste center of a single institution.

We reviewed self-reported and measured outcomes of gustatory and olfactory function using patient data including clinical characteristics, including age, sex, smoking history, and medical history.

**Results:**

We found that the subjective recognition of gustatory dysfunction does not correlate with the objective diagnosis of gustatory dysfunction based on the measured results of gustatory function tests. Subjective gustatory dysfunction, however, was correlated with subjective olfactory dysfunction. Among clinical and demographic characteristics, age and sex were significantly associated with measured gustatory function outcomes.

**Conclusion:**

We suggest that subjective gustatory dysfunction underestimates objective dysfunction and recommend that older men with diminished olfactory function undergo gustatory function testing regardless of their self-reported gustatory function status.

## Background

Older adults have become the fastest-growing segment of the population. In the United States, it is estimated that the number of adults older than 55 years will constitute 29% of the population by 2030 [1]. In geriatrics, sensory loss is an important issue, particularly because of its impact on overall health and well-being [2]. Gustation and olfaction are vital to the perception and experience of daily life, and impaired gustatory and olfactory function results in decreased appetite, nutritional deficits, emotional distress, and even increased mortality [3].

Olfactory dysfunction and the evaluation of olfactory function in geriatrics have been actively studied. Structural changes in olfactory system explain the natural decline in olfactory function with age [4]. Also, olfactory dysfunction has been associated with various systemic diseases, and evaluation of olfactory function in geriatrics is now accepted as an important screening tool in the diagnosis of various diseases, such as Alzheimer’s disease and Parkinson’s disease [5, 6]. As subjective recognition of olfactory dysfunction correlates with objective diagnosis of olfactory dysfunction [7], olfactory function testing is usually recommended for patients with subjective olfactory dysfunction.

Compared with olfactory dysfunction, gustatory dysfunction and its evaluation have been less studied and underestimated in geriatrics. Physiologic changes associated with the aging process, drug use, zinc deficiency, oral pathologies, and various systemic diseases have been found to be associated with taste disorders in the geriatrics [8]. Perception of each of the five main flavors (salty, sweet, sour, bitter, and umami taste) enables an individual to evaluate quality of the food consumed, and decreased taste function have negative consequences on the health of geriatrics [8]. However, in contrast to the availability of olfactory function tests, the lack of a universally accepted standard method for taste function assessment limits the evaluation of gustatory function in geriatrics [9].

It has been suggested that severe gustatory dysfunction is an independent predictor of cognitive impairment among older adults [10]. However, recent studies have demonstrated a lack of concordance between self-reported and objectively measured gustatory dysfunction outcomes. Bernstein et al. [3] found no association between self-reported and measured gustatory dysfunction outcomes; however, their study included only salty and bitter tastes, omitting sweet, sour, and umami tastants in their assessment of gustatory function. Wolf et al. [11] reported that patients’ self-reported subjective gustatory dysfunction showed no correlation with the results of gustatory function tests, but they only evaluated the correlation between the scores of subjective discomfort and objective gustatory function test; they did not compare the association between normal and impaired gustatory function. Therefore, we investigated whether patient-reported gustatory dysfunction could be a sufficient clinical indication for administering conventional gustatory function testing in older patients.

## Methods

### Study design

We retrospectively enrolled individuals aged ≥ 60 years who visited our smell/taste center for evaluation of olfactory and gustatory function between January 2018 and April 2021. All study protocols were performed in accordance with the Declaration of Helsinki, and ethical approval for the study was obtained from the Institutional Review Board of the Chung-Ang University Hospital. We excluded patients with any past or current diagnosis of cognitive dysfunction, acute or chronic rhinosinusitis, or a history of nasal surgery. We obtained demographic and clinical data, including previous medical history, from medical records.

### Gustatory and olfactory function tests

We defined self-reported gustatory/olfactory dysfunction by a positive answer to the following question: “Have you had a problem with taste/smell in the past 12 months?” We based objective diagnoses of gustatory dysfunction on the measured outcomes of a gustatory function testing protocol that was developed and validated in the Korean population [12]. Briefly, the testing protocol consisted of 30 taste solutions (six concentrations of each of the five tastants: sweet (sucrose with a concentration ranging from 0.0048 to 0.1563 g/mL), bitter (quinine hydrochloride; 0.00005–0.0016 g/mL), salty (sodium chloride; 0.0006–0.0192 g/mL), sour (citric acid; 0.0002425–0.00781 g/mL), and umami (monosodium glutamate; 0.002–0.064 g/mL). We placed a single drop (approximately 40 µL) of tastant on the middle part of the anterior one-third of the tongue, and between the drops, we told patients to rinse their mouths with tap water. For each taste, we defined the detection threshold as the lowest concentration of test solution that could be perceived by patients as any taste, and the test was repeated two times. We defined a ‘taste score,’ which we used to evaluate the overall gustatory function as the summed number of detected and correctly recognized taste thresholds, and patients with a recognition taste score < 12 were regarded as having impaired gustatory function [12]. We performed electrogustometry (EGM; using TR-06 from Rion Co., Ltd., Tokyo, Japan) following a previously reported protocol with minor modifications [13]. We applied the electric stimulus with a bipolar electrode, which we placed on the anterior and posterior regions of the tongue on the right side. We applied stimuli starting at 6 dB (1.5 mA) and went up to 40 dB for 0.5 s, and we measured the minimal level that each patient perceived as a threshold outcome. We considered a threshold > 30 µA to be pathological [14]. We measured olfactory function using the YSK olfactory function test (RHICO Medical Co., Seoul, Korea), which contains three subsets: threshold, discrimination, and identification [6]. We calculated the sum of the three test scores as the threshold–discrimination–identification (TDI) score (total score: 1–36). We diagnosed patients with TDI scores < 21 with olfactory dysfunction.

### Statistical analysis

We performed statistical analyses using SPSS Statistics for Windows, version 19.0 (IBM Corp., Armonk, NY, USA). We present descriptive data as means ± standard deviations. We performed χ^2^ analysis for intergroup comparisons. The differences between the two groups, i.e., patients reporting a subjective gustatory dysfunction versus those not reporting such dysfunction, were analyzed using independent t-test. We performed univariate and multivariate regression analyses to identify factors associated with the presence of measured gustatory dysfunction, and *p*-values lower than 0.05 were considered to indicate statistically significant differences.

## Results

Characteristics of enrolled patients are shown in Table 1. A total of 138 patients, 66 men and 72 women, were included in the study. The mean age was 67.85 ± 6.98 (range, 60–85) years. Thirty-six patients (25.9%) self-reported subjective gustatory dysfunction, and 31 (22.3%) were objectively diagnosed with gustatory dysfunction. The mean EGM threshold was 2.90 ± 2.68 µA in the anterior tongue and 11.84 ± 7.38 µA in the right posterior tongue. Sixty-four patients (46%) had subjective self-reported olfactory dysfunction. The mean YSK_TDI score of the patients enrolled was 17.38 ± 6.15 (Table 1). The measured detection threshold scores for sweet, bitter, and umami tastes were, respectively, 2.87 ± 1.27, 4.48 ± 0.93, and 5.08 ± 0.98 in patients without subjective gustatory dysfunction. Corresponding threshold scores in patients with subjective gustatory dysfunction were 3.03 ± 1.27, 4.61 ± 0.76, and 5.15 ± 0.46 (Table 2). The measured sweet and bitter taste recognition threshold scores were 2.69 ± 1.075 and 2.91 ± 1.09, respectively, in patients without subjective gustatory dysfunction; and 2.92 ± 1.07 and 3.03 ± 1.08 in patients with subjective gustatory dysfunction (Table 2). However, none of the measured detection or recognition threshold scores were significantly associated with the presence of subjective gustatory dysfunction.

**Table 1:** Patient characteristics

Variable	Value
Number	138
Sex	
Male : female	66 : 72
Age (years)	
Mean ± SD	67.85 ± 6.98
Range	60–85
Smoking status	
Nonsmoker, *n* (%)	72 (51.8)
Smoker, *n* (%)	66 (47.5)
Patients with hypertension, *n* (%)	34 (24.5)
Patients with diabetes mellitus, *n* (%)	5 (3.6)
Patients with subjectively recognized gustatory dysfunction, *n* (%)	36 (25.9)
Patients with objectively diagnosed gustatory dysfunction, *n* (%)	31 (22.3)
Electrogustometry, anterior (µA)	2.90 ± 2.68
Electrogustometry, posterior (µA)	11.84 ± 7.38
Patients with subjectively recognized olfactory dysfunction, *n* (%)	64 (46)
YSK_TDI	17.38 ± 6.15

*SD* Standard deviation, *YSK_TDI* YSK_threshold-discrimination-identification

**Table 2:** Chemical gustometry test scores according to the presence of subjective gustatory dysfunction

Taste detection threshold	With subjective gustatory dysfunction (Mean ± SD)	Without subjective gustatory dysfunction (Mean ± SD)	*P*-value
Sweet	3.03 ± 1.27	2.87 ± 1.27	0.527
Bitter	4.61 ± 0.76	4.48 ± 0.93	0.435
Salty	4.17 ± 0.91	4.34 ± 0.87	0.324
Sour	4.44 ± 0.87	4.60 ± 8.84	0.353
Umami	5.15 ± 0.46	5.08 ± 0.98	0.375
Taste recognition threshold			*P*-value
Sweet	2.92 ± 1.07	2.69 ± 1.075	0.286
Bitter	3.03 ± 1.08	2.91 ± 1.09	0.136
Salty	2.03 ± 1.00	2.33 ± 1.07	0.148
Sour	2.92 ± 1.18	3.01 ± 1.22	0.694
Umami	2.89 ± 1.05	3.05 ± 1.85	0.875

*SD* Standard deviation

**Fig. 1:**
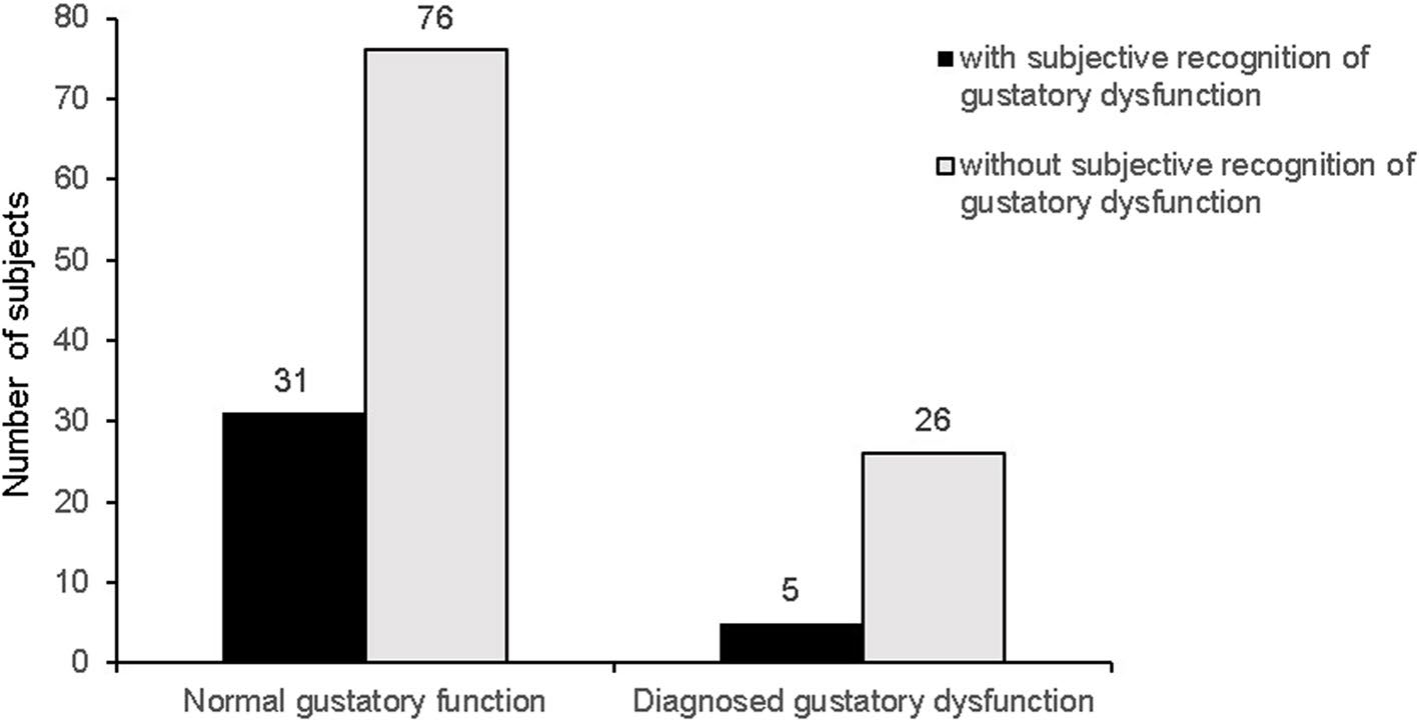
Distribution of subjectively recognized gustatory dysfunction between individuals with objectively normal and impaired gustatory function (χ^2^ = 2.056, *p* = 0.152)

We performed χ^2^ analysis to determine if subgroups differed in terms of subjective discomfort and the outcome of chemical gustatory function test; 83.8% (26 of 31) of patients with objectively diagnosed gustatory dysfunction did not have subjective recognition of gustatory dysfunction (χ^2^ = 2.056, *p* = 0.152) (Fig. 1). Multivariate logistic analysis revealed that old age, male gender, and subjective recognition of olfactory dysfunction were significantly associated with objective diagnosis of gustatory dysfunction based on the gustatory function test (Table 3).

**Table 3:** Results of multivariate logistic regression analysis of clinical characteristics, including subjectively recognized gustatory dysfunction and the presence of objectively diagnosed gustatory dysfunction

Variable	Univariate		Multivariate	
	Odds ratio (95% CI)	*P* value	Odds ratio (95% CI)	*P*-value
Age	1.059 (1.001–1.121)	0.048	1.099 (1.026–1.177)	0.007
Sex	2.893 (1.243−6.737)	0.014	0.213 (0.079–0.574)	0.002
Smoking history	0.400 (0.239−1.931)	0.058		
Subjective gustatory dysfunction	2.121 (0.747−6.027)	0.158		
EGM, anterior	1.030 (0.888−1.194)	0.695		
EGM, posterior	1.055 (0.999−1.114)	0.053		
Subjective olfactory dysfunction	3.909 (1.552–9.843)	0.003	4.973 (1.797–13.764)	0.002
Objectively diagnosed olfactory dysfunction	0.882 (0.366–2.124)	0.779		
YSK-TDI	0.986 (0.920−1.055)	0.677		

*CI* Confidence interval, *EGM* Electrogustometry, *YSK-TDI* YSK_threshold-discrimination-identification

**Fig. 2:**
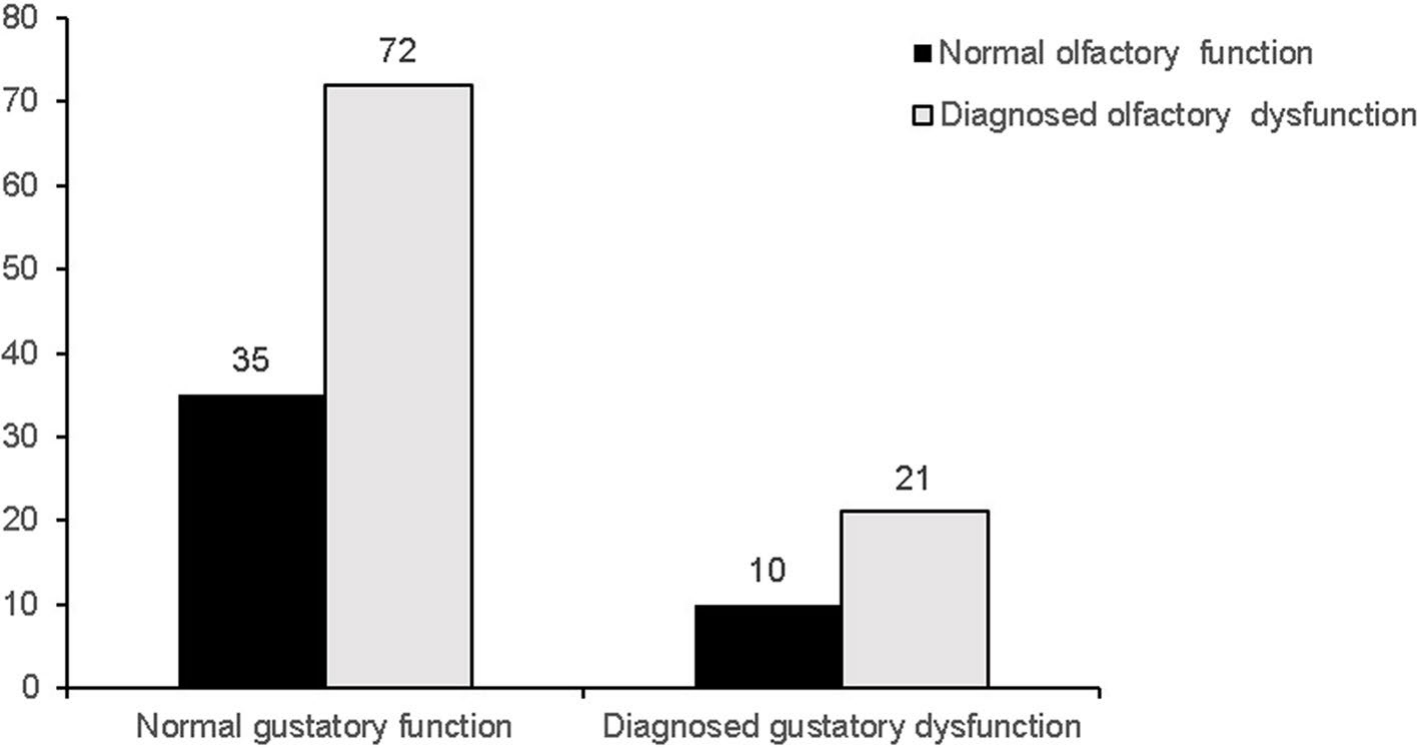
Distribution of objectively diagnosed olfactory dysfunction between individuals with objectively normal and impaired gustatory function (χ^2^ = 0.079, *p* = 0.779)

Finally, we evaluated the prevalence of olfactory dysfunction in patients with and without gustatory dysfunction. We found that 67.3% (72 of 105) of patients without gustatory dysfunction were diagnosed with olfactory dysfunction, and 67.7% (21 of 31) of patients with gustatory dysfunction were also diagnosed with olfactory dysfunction (χ^2^ = 0.0079, *p* = 0.779) (Fig. 2).

## Discussion

Chemosensory function such as olfaction and gustation tend to be underrecognized although they are critical contributors to overall health and quality of life specifically impacting patients’ nutritional status and psychological well-being [15]. We found that subjective recognition of gustatory dysfunction did not correlate with objectively diagnosed gustatory dysfunction, and we suggest that gustatory function testing should be considered regardless of symptoms, especially for older men.

Compared with gustatory dysfunction, many more studies have investigated olfactory dysfunction, and it has been reported that subjective recognition of olfactory dysfunction correlates with measured olfactory outcomes. We also found that subjective recognition of olfactory dysfunction correlated with objective diagnosis of olfactory dysfunction, and the correlation was consistent among patients with impaired cognitive function [7].

It has been reported that olfactory dysfunction leading to a perceived gustatory impairment is common due to the complex interaction of chemosenses [15]. In contrast to our expectations, however, the presence of objectively diagnosed olfactory dysfunction was not significantly associated with the presence of objectively diagnosed gustatory dysfunction (Fig. 2). We suggest that future study on a larger population is needed to elucidate the relationship between objective olfactory and gustatory dysfunction in geriatrics.

Olfactory dysfunction has been accepted as an early sign of various neurodegenerative diseases, and early recognition of olfactory dysfunction has become more important, especially in geriatrics, for the early diagnosis and treatment of these neurodegenerative diseases [8]. Although there is limited supporting evidence, gustatory dysfunction has been regarded as an early sign of neurodegenerative diseases, such as dementia and Parkinson’s disease [8]. We did not find that self-reported gustatory dysfunction was sufficient for administering a conventional objective gustatory function assessment; however, we recommend gustatory function testing for all older patients with subjective olfactory dysfunction.

In our study, EGM outcomes were not significantly associated with the diagnosis of gustatory dysfunction which was based on chemical gustatory function test (Table 3). Considering that the mean EGM threshold was within normal limits, our findings should be interpreted with caution. EGM is used as a clinical tool for estimating taste detection thresholds; however, EGM is limited by low sensitivity and specificity [14]. EGM findings depend on the condition of the electrode, and it only detects sourness rather than reflecting the overall responses to all tastants. Therefore, the relationship between self-reported gustatory dysfunction and EGM findings was inconclusive in this study, and future large-scale research is warranted.

A population study found gustatory dysfunction in approximately 20% of adults in the general population [16]. In another study, severe gustatory dysfunction was reported in 14.8% of the population older than 55 years, which was a much higher prevalence than that for severe olfactory dysfunction, which was reported to affect 2.7% of the same population [1]. The prevalence of gustatory dysfunction is suspected to be higher in older populations. Therefore, we suggest that gustatory function assessments be widely administered to older patients even if they do not report subjective sensory loss.

Unlike olfactory function tests, standardized clinical tests of gustatory function are rarely available, and literature on the prevalence of gustatory dysfunction is scarce. Commonly applied gustatory function tests are chemical gustatory function tests that utilize either solutions or strips. Representative salty, sweet, bitter, and sour tastes in various concentrations are used. In our study, we performed chemical solution tests with five tastants (including umami) and six gradient concentrations. In a preliminary study, a spray test was applied [17]. EGM could be considered; however, EGM results themselves are not conclusive. A chemical gustatory function test should be applied, and EGM could be utilized as an additional tool in a very limited cases, such as when the result of chemical gustatory function test is doutful. Lifestyle factors, such as consumption of a Western-style diet, could result in variations in chemosensory abilities [18], and culture-specific gustatory function tests with corresponding normal ranges should be established and implemented for the proper diagnosis of gustatory dysfunction among older adults.

This study had some limitations. First, it was conducted at a single tertiary hospital. Therefore, our results should be validated by a large population-based study. Second, although we considered several factors that might influence subjective and objective gustatory function, such as smoking and concurrent medical diseases, other potentially associated factors such as cognitive dysfunction, body mass index, and medication history need to be evaluated [10]. Further research investigating these factors is warranted.

## Conclusion

Objective gustatory test may not faithfully reflect self-reported outcomes due to a low correlation between the two measures. Therefore, we recommend to perform chemical gustatory function test even though the patients do not complaint subjective gustatory dysfunction, especially in male geriatrics who require olfactory function assessments.

## Data Availability

The datasets generated and/or analyzed during the current study are available from the corresponding author on reasonable request.

## Abbreviations

EGM: Electrogustometry
TDI: Threshold-discrimination-identification
